# Dacryocystitis Secondary to Orbital Mesh Implant Impingement: A Rare Etiology

**DOI:** 10.7759/cureus.66001

**Published:** 2024-08-02

**Authors:** Arulvignesh M, Lakshmi Rathan A C, Vivek Narayanan, Prashanthi Gurram, Abinaya Subramanian

**Affiliations:** 1 Department of Oral and Maxillofacial Surgery, SRM Kattankulathur Dental College and Hospital, SRM Institute of Science and Technology (SRMIST), Chengalpattu, IND

**Keywords:** dacryocystitis, dacryocystography, dacryocystorhinostomy, nasolacrimal apparatus, orbital mesh

## Abstract

A 22-year-old male patient reported swelling in relation to the right eye and developed recurrent purulent discharge and epiphora following a reconstructive traumatic orbital floor fracture repair two years ago. Radiographic investigation and surgical exploration reveal obstruction of the lacrimal apparatus at the lacrimal sac level due to over-extension/migration of the orbital floor mesh. The migration of the mesh was probably due to the extension of the mesh medially into the paranasal region.

## Introduction

An inflammatory condition of the nasolacrimal sac is known as dacryocystitis. It is usually due to obstruction in the nasolacrimal duct, which causes the tears to stagnate in the lacrimal apparatus.

Dacryocystitis is a potentially dangerous illness marked by acute infection and inflammation of the lacrimal apparatus, and it is defined as "a medical urgency that is clinically characterized by the rapid onset of pain, erythema, and swelling, classically below the medial canthal tendon, with or without preexisting epiphora, mainly resulting from the acute infection of the lacrimal sac and perisac tissues" [1].

## Case presentation

A 22-year-old male reported to our Department of Oral and Maxillofacial Surgery with a complaint of pain and swelling in relation to the right lower eyelid region for the past two weeks. The patient was apparently normal for two weeks, after which he developed a small swelling in relation to the lower eyelid. The swelling was initially small and gradually increased to its current size, accompanied by vague pain. He had consulted multiple hospitals for the same and was prescribed basic analgesics, antibiotic eye drops, and oral antibiotics. Despite treatment, his symptoms rapidly worsened.

The patient had a previous history of trauma due to a road traffic accident and was diagnosed with a fronto-naso-orbito-ethmoidal fracture, for which he was treated with open reduction and internal fixation, orbital exploration, and orbital floor reconstruction under general anesthesia two years ago.

**Figure 1:**
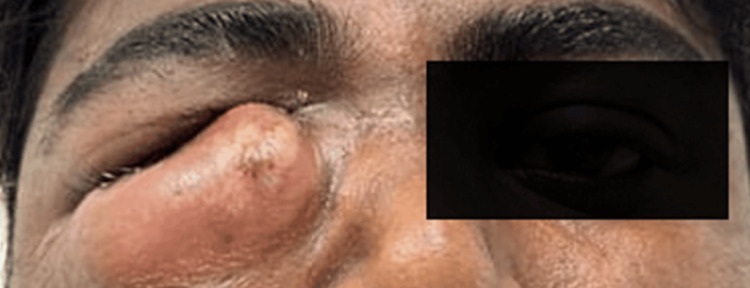
Extraoral picture showing swelling in relation to the right lower eyelid covering the entire eye

On examination, the patient had a solitary swelling measuring 6 cm x 4 cm in the right lower eyelid region. An erythematic, voluminous mass was localized just inferior to the medial canthus of the right eye, covering most of the eye and causing obstruction to vision (Figure 1). On palpation, it was tender, fluctuant, and warm. The patient had a skin fistulous communication and punctal leakage of purulent discharge upon applying pressure. The patient did not suffer any recent trauma, associated fever, or signs of conjunctivitis. Vision was normal, and extraocular eye movements were free and full. An ophthalmic opinion was obtained, and an implant site infection was suspected.

Following this, the patient was immediately admitted and started on intravenous antibiotics (Inj cefotaxime 1 g, Inj metrogyl 500 mg), and a pus culture was sent, which turned out to be sterile.

High-resolution computed tomography was performed, which revealed medial impingement of the orbital mesh over the nasolacrimal duct apparatus; obvious obliteration was noted. A treatment plan for orbital exploration and orbital mesh removal under general anesthesia was suggested.

The patient was taken up for surgery, intubated nasoendotracheally, and general anesthesia was induced following standard protocols. The patient was painted, prepared, and draped. Through the existing scar tissue at the previous midtarsal incision site, a blunt dissection was carried out to reach the bony septum, orbital rim, and orbital floor. A well-adapted mesh and floor were noted; while attempting to remove the mesh, the operating surgeon encountered difficulty in removing it from the medial aspect because the medial flange was overextended and had fused with the medial nasal bone, compromising the patency of the nasolacrimal duct. The fused part of the mesh was removed in two pieces. A thorough metrogyl wash and irrigation were given, and layer-wise closure was achieved using Vicryl 4-0 and Ethilon 4-0 (Ethicon, Somerville, NJ, USA). The patient was extubated, and recovery was uneventful. Sutures were removed after one week, and healing was satisfactory.

One month postoperatively, the patient returned for review with swelling in the same region, indicating a rupture of the soft tissue duct and the need for repair. The patient was then referred to an ophthalmic center for nasolacrimal duct repair.

## Discussion

An inflammatory condition of the nasolacrimal sac is known as dacryocystitis. It is usually due to obstruction in the nasolacrimal duct, which causes tears to stagnate in the lacrimal apparatus. Clinically, dacryocystitis is recognized by swelling and inflammation of the lacrimal sac at the inferomedial canthus [2].

A better understanding of dacryocystitis and its possible multilayer involvement arises from knowledge of the anatomy of the apparatus and the flow of tears. When tears are produced by the lacrimal gland, they normally start to flow. Tears continue to lubricate the eye until they are gathered in the superior and inferior puncta and drained into the common canaliculus. They then proceed to enter the lacrimal sac by passing through the Rosenmuller valve. Following the collection of tears in the lacrimal sac, the tears travel via the nasolacrimal duct, Hasner valve, and ultimately to the nasal cavity.

Obstruction, stagnation, and infection are characteristic sequelae of dacryocystitis, causing edema and associated discharge. Communicating fistula formation between the skin and the lacrimal sac is an unusual sequela. Of the patients in the cited case series, 5.6% had a fistula, of which 83.3% had the lacrimal abscess burst spontaneously and 16.7% as a result of incision and drainage [3,4]. However, infective sinusitis, foreign bodies, facial traumas, and direct microbial inoculation are quite common etiologies. Methicillin-sensitive *Staphylococcus aureus* is the most commonly isolated germ from bacterial cultures of the purulent discharge (collected at the sac, fistula, or abscess surgical incision), followed by *Streptococcus pneumoniae* [5]. However, in our case, the culture sensitivity turned out to be sterile pus with no growth; this could be because the patient was loaded with antibiotics in various hospitals.

Traumatic dacryocystitis is often due to a fracture of the bone surrounding the nasolacrimal duct, most often associated with fronto-nasoorbital-ethmoidal fractures. An untreated or incompletely treated blow-out fracture, or immediate tissue damage incurred with traumatic naso-orbital, orbital blow-out, and paranasal-maxillary fractures, can commonly lead to lacrimal obstruction, loss of the lacrimal pump mechanism, and nonspecific dacryocystitis [6,7]. Additionally, a few cases have been reported with implant loosening and migration of the mesh, subsequently causing an obstruction. 

**Figure 2:**
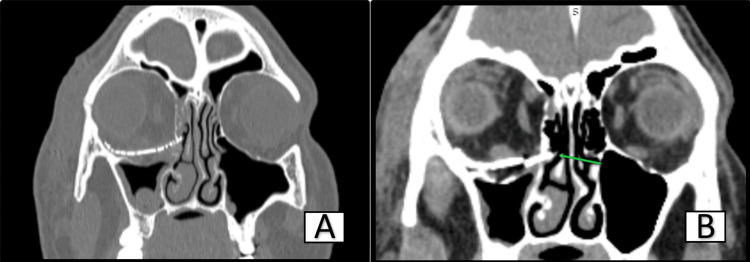
HRCT coronal section showing overextension of orbital mesh medially (green arrow) HRCT: High-resolution computed tomography

**Figure 3:**
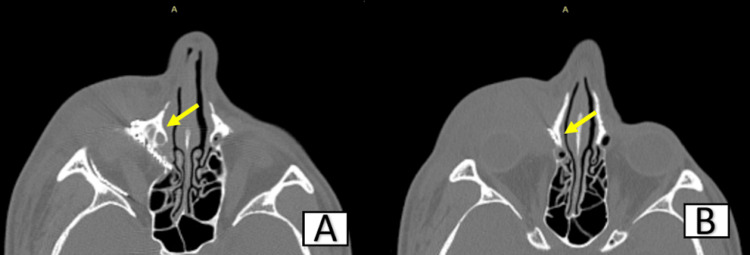
Axial section showing overextension of the orbital mesh medially (arrows represent the lacrimal duct being impinged)

In this case, lacrimal obstruction was caused by an orbital mesh placed after a successful reconstruction of a blow-out fracture repair two years ago, which was clearly evident in the high-resolution computed tomography (Figures 2-3). Damage to adjacent periorbital and paranasal structures may result from an overextended or improper reconstruction. Failure to place the mesh subperiosteally against the underlying bone may predispose it to migration. The orbital mesh should be placed subperiosteally, smoothing the edges to avoid damage to adjacent vital structures [8-10].

## Conclusions

Though dacryocystitis is usually due to obstruction of the nasolacrimal duct, this can also be iatrogenic, especially in cases of fronto-orbital-ethmoidal fractures and medial wall of orbit fractures. Proper preoperative and postoperative assessment, avoiding overextension of orbital meshes, virtual surgical planning, dynamic navigation surgeries in orbital floor repair, and patient-specific implants can prevent such postoperative complications.
